# P040 Takayasu arteritis mimicking ANCA-associated vasculitis and presenting with acute myocardial dysfunction in a young woman

**DOI:** 10.1093/rap/rkag113.070

**Published:** 2026-11-03

**Authors:** Bodhibrata Banerjee, Jagannath De, Anindita Sinha, Sanjay Jain, Varun Dhir

**Affiliations:** Rheumatology Wing, Department of Internal Medicine, Postgraduate Institute of Medical Education and Research, Chandigarh, Chandigarh, India; Rheumatology Wing, Department of Internal Medicine, Postgraduate Institute of Medical Education and Research, Chandigarh, Chandigarh, India; Department of Radiodiagnosis, Postgraduate Institute of Medical Education and Research, Chandigarh, Chandigarh, India; Rheumatology Wing, Department of Internal Medicine, Postgraduate Institute of Medical Education and Research, Chandigarh, Chandigarh, India; Rheumatology Wing, Department of Internal Medicine, Postgraduate Institute of Medical Education and Research, Chandigarh, Chandigarh, India

## Abstract

**Introduction:**

Takayasu arteritis (TAK) is a large-vessel vasculitis that predominantly affects young women. Cardiac involvement occurs in roughly half of patients over the disease course and is a leading cause of morbidity and mortality, yet acute heart failure as the presenting feature remains under-recognised. Rarely, its constitutional, ENT and pulmonary manifestations can closely mimic ANCA-associated vasculitis, delaying diagnosis. We describe a young woman whose fever, haemoptysis and epistaxis initially suggested ANCA-associated vasculitis, but who was ultimately found to have large-vessel TAK complicated by acute myocardial dysfunction—illustrating two diagnostic pitfalls in a single presentation.

**Case description:**

A 22-year-old woman presented with one month of fever (101–102 °F), initially intermittent and later with 2–3 daily spikes, one week of cough with streaky haemoptysis, and four days of progressive exertional dyspnoea with orthopnoea. She also reported a single self-limiting episode of epistaxis. There were no features of connective tissue disease (no arthritis, Raynaud’s, oral ulcers, photosensitivity or sicca). She had recently been found hypertensive.

On examination she was pale, with blood pressure 160/110 mmHg (equal in both arms), a feeble left carotid pulse, a left renal bruit, a left parasternal heave and bibasal crepitations. Investigations showed normocytic anaemia, neutrophilic leucocytosis and raised inflammatory markers (ESR 95 mm/h; CRP 110 mg/L). NT-proBNP was markedly elevated (13,183 pg/mL) with mildly raised troponin-T; renal impairment was mild and transient. ANA, ENA, ANCA (MPO/PR3), antiphospholipid antibodies, IgG4 and myositis profile were negative or normal. An infective screen—blood and urine cultures, Mantoux, viral serologies and procalcitonin—was unremarkable, and urinalysis showed no active sediment with normal 24-hour protein.

Transthoracic echocardiography revealed global hypokinesia (LVEF 25–30%) with moderate mitral regurgitation. Carotid Doppler demonstrated diffuse circumferential mural thickening of both common carotid arteries. CT angiography showed circumferential mural thickening of the aorta, bilateral ostioproximal renal artery stenosis, and thrombosis of the left common carotid and superior mesenteric arteries, with bilateral pleural effusions and pulmonary oedema.

A diagnosis of Takayasu arteritis (ITAS 8) with acute myocardial dysfunction and secondary renovascular hypertension was made. She received pulsed methylprednisolone, then oral prednisolone (1 mg/kg) with intravenous cyclophosphamide, plus heart-failure therapy, achieving symptomatic improvement.

**Discussion:**

This case illustrates two diagnostic pitfalls. First, the early picture—fever, haemoptysis, epistaxis and pulmonary infiltrates—closely mimicked ANCA-associated vasculitis (AAV). Several features redirected the diagnosis: ANCA (MPO/PR3) negativity, absence of glomerulonephritis (bland sediment, normal proteinuria), renovascular rather than glomerular renal involvement, and large-vessel imaging demonstrating aortic and branch-vessel arteritis. In a young woman, this constellation should prompt vascular imaging before anchoring on AAV.

Second, acute myocardial dysfunction is an under-recognised presenting feature of TAK, and its cause is characteristically multifactorial. Recognised mechanisms include renovascular and aortic (afterload) hypertension causing pressure-overload failure; myocarditis, which can precipitate reversible heart failure independent of hypertension or valve disease; ischaemia from coronary arteritis (typically ostial) or coronary microvascular dysfunction; volume overload from aortic regurgitation with secondary mitral regurgitation; and pulmonary arteritis causing pulmonary hypertension with right-heart failure. Systemic inflammation and increased arterial stiffness contribute mechanically.

In our patient, dysfunction was most plausibly dominated by renovascular-hypertensive afterload (bilateral renal artery stenosis; BP 160/110 mmHg), with possible concurrent myocarditis given the acute inflammatory context and mildly raised troponin. The moderate mitral regurgitation was functional, secondary to ventricular dilatation, rather than primary valvular disease; aortic regurgitation was absent. Epicardial and microvascular coronary disease were not formally assessed.

The principal limitation was the absence of cardiac MRI—the recommended non-invasive modality to characterise myocardial inflammation and an alternative to endomyocardial biopsy—which would have clarified the dominant mechanism and aided prognostication. Prompt immunosuppression combined with heart failure therapy nonetheless achieved rapid clinical improvement, consistent with the reversibility of inflammatory myocardial involvement in TAK.

**Key learning points:**

• First, Takayasu arteritis can present acutely with heart failure: acute myocardial dysfunction may be the index manifestation rather than a late complication. Large-vessel vasculitis therefore warrants consideration in any young patient—particularly a young woman—with unexplained ventricular dysfunction and a systemic inflammatory response.

• Second, the early constitutional, ENT and pulmonary features of TAK can closely mimic ANCA-associated vasculitis. ANCA (MPO/PR3) negativity, the absence of glomerulonephritis, renovascular rather than glomerular renal involvement, and large-vessel imaging are the discriminating features; vascular imaging should be pursued before anchoring on a small-vessel diagnosis.

• Third, myocardial dysfunction in TAK is characteristically multifactorial, and identifying the dominant mechanism directs treatment. Contributors include renovascular and aortic (afterload) hypertension, myocarditis, ischaemia from coronary arteritis or microvascular dysfunction, valvular disease (chiefly aortic regurgitation, with secondary mitral regurgitation), and pulmonary arteritis causing right-heart strain. Separating reversible inflammatory dysfunction from fixed structural or ischaemic causes changes management.

• Fourth, cardiac MRI is the key modality for characterising the mechanism and is a meaningful gap when unavailable; nonetheless, prompt immunosuppression combined with heart-failure therapy can achieve rapid clinical improvement, consistent with the reversibility of inflammatory myocardial involvement. Early recognition and treatment of the underlying vasculitis—not heart-failure management alone—is central to outcome.

